# Self-reported functional, communicative, and critical health literacy on foodborne diseases in Accra, Ghana

**DOI:** 10.1186/s41182-018-0097-6

**Published:** 2018-05-15

**Authors:** Sangeeta Gupta, Raymond Asare Tutu, John Boateng, Janice Desire Busingye, Sathya Elavarthi

**Affiliations:** 1 Department of Public Health, Delaware State University, Dover, DE 19901 USA; 2 Global Societies Program, Delaware State University, 1200 North DuPont Highway, Dover, DE 19901 USA; 3 School of Continuing and Distance Education, University of Ghana, Legon, Accra Ghana; 4 Kampala International University, P.O Box 20000, Kampala, Uganda; 5 Department of Agriculture and Natural Resources, Delaware State University, Dover, DE 19901 USA

**Keywords:** Health literacy, Foodborne diseases, Urban slums, Tropical medicine, Ghana

## Abstract

**Background:**

Although substantial progress has been made in reducing total mortality resulting from foodborne diseases, diarrheal illness are still the second most common illnesses among children. In Ghana, foodborne diseases have consistently been among the top 20 causes of outpatient illness over the last couple of decades. This study, therefore, examines health literacy on foodborne diseases and the relative effects of health literacy on self-rated health.

**Methods:**

Foodborne diseases are major causes of morbidity and mortality globally. A mixed-method approach was used for this study. A survey questionnaire and an in-depth interview guideline were administered to samples of 401 and 30 individuals, respectively. We undertook reliability and validity analyses. ANOVA and chi-square tests were undertaken to assess bivariate association between health literacy and demographic variables as well as health status. Ordinal logistic regression models were used to examine the relative effects of health literacy on self-rated health status controlling for individual characteristics.

**Results:**

The instrument was internally consistent (Cronbach alpha = 0.744) and valid. On health literacy, 40% of the respondents reported not to require help when they are given information on foodborne diseases to read by a doctor, nurse, or pharmacist. Approximately 60% of respondents need help with completing or filling out hospital documents. Educational level was found to be positively related to functional health literacy. Ordinal logit regression models showed that health literacy is a predictor of self-rated health after controlling for demographic variables.

**Conclusion:**

Functional literacy is relatively low in the community. There is a positive association between educational level and functional health literacy. The study has also demonstrated the direct positive relationship between health literacy and health status controlling for covariates. Subsequent studies will need to examine multiple level dimensions of health literacy with direct link between specific foodborne diseases and health literacy.

**Electronic supplementary material:**

The online version of this article (10.1186/s41182-018-0097-6) contains supplementary material, which is available to authorized users.

## Background

### Introduction

Globally, foodborne diseases are a leading cause of morbidity and a major reason for in-patient and out-patient hospitalization with adverse impact on economic and development agenda [1, 2]. An estimated 250 different foodborne illnesses result from the consumption of food and water adulterated with pollutants and pathogens like parasites, bacteria, and viruses [3–5]. Although significant progress has been made in the reduction of global diarrheal deaths, there is still much work to be done [6, 7]. The World Health Organization (WHO) estimates show that there are significant geographic variations in the global burden of foodborne illnesses given mortality estimates across sub-regions [8]. Africa experienced the highest burden per population, followed by South East Asia and Eastern Mediterranean, primarily due to inadequate food safety knowledge [9–11].

In Ghana, foodborne diseases are among the leading causes of morbidity and mortality. In 2016, diarrhea was fourth of the top 10 diseases reported [12, 13]. A study of trends and patterns of foodborne diseases reveals differences in the number of cases that are reported in hospitals. For example, in an attempt to assess the patterns of foodborne diseases at the Ridge Hospital in Accra, Ghana, Osei-Tutu and Anto [14] found that the foodborne diseases commonly reported were typhoid fever, viral hepatitis, dysentery, and cholera.

There are wide-ranging sources of foodborne diseases in Ghana. While it has been affirmed that the home environment is a breeding haven for foodborne pathogens partly due to poor personal hygienic practices [15], other food-handling outlets contributing to foodborne diseases are restaurants, food vendors, local food stands, and institutions, like schools with feeding facilities, hospitals, as well as prisons, and these food outlets are responsible for over 70% of all traceable foodborne illnesses in the country [16–20].

Clearly, foodborne diseases are a major public health headache for Ghana. While a substantial amount of research has been undertaken to understand factors responsible for foodborne diseases—knowledge, attitudes, and practices of food-handlers; knowledge of consumers regarding food safety components and practices; and the patterns of foodborne diseases—there is little information on health literacy of urban poor consumers regarding foodborne diseases. This study, therefore, attempts to explore the functional, interactive/communicative, and critical health literacies on foodborne diseases among the urban poor in Accra, Ghana. Specifically, the study asks the following questions: (1) Are there associations between health literacy and individual demographic characteristics? (2) Is health literacy significantly associated with self-rated health status in James Town?

### Health literacy as a conceptual and practical tool

Studies have shown the importance of health literacy in maintaining or improving population health [21–23] as well as the linkages between health literacy and individual/demographic characteristics [24, 25]. For example, health literacy is associated with racial and education-related health disparities primarily because health literacy is normally affected by general literacy [24–29]. Health literacy is a multidimensional concept and, consequently, has multiple definitions. For example, while the WHO [4] defines health literacy as “the cognitive and social skills which determine the motivation and ability of individuals to gain access to, understand and use information in ways which promote and maintain good health,” the Institute of Medicine [30] defines it as “the degree to which individuals have the capacity to obtain, process, and understand basic health information and services needed to make appropriate health decisions.” Although these definitions may differ in their operationalization, both of them emphasize individual capabilities that allow a person to get and use new health information. While health literacy goes beyond individual capabilities [31], a number of seminal health literacy tools (e.g., Rapid Estimate of Adult Literacy in Medicine (REALM-R), the Test of Functional Health Literacy in Adults (TOFHLA), Health Activities Literacy Scale, and the Newest Vital Signs) have been developed to measure individual capacity [32–35]. These instruments have, however, been critiqued for being lengthy and sometimes culturally inappropriate [26, 36].

Subsequent to these health literacy instruments, others have been developed for specific context and to measure specific health literacy definitions [26, 37, 38], as well as disease-specific measurements [39, 40]. Chinn and McCarthy’s [37] health literacy tool, a self-report items instrument, is largely premised on Nutbeam’s [41] health promotion-oriented model which discusses three health literacy typologies: functional, interactive (communicative), and critical health literacies. Functional health literacy refers to the individual’s basic ability to read and write as well as the possession of basic knowledge of health conditions. Interactive health literacy denotes social skills that can be used to acquire information and deduce meaning from different communication forms while applying it to various varying conditions. Critical health literacy indicates a higher level of social and cognitive skills that enables the individual to critically examine health information thereby able to exercise control over one’s life course. These typologies, according to Sørensen et al. ([42] p.4–5), “represent levels of knowledge and skills that progressively support greater autonomy and personal empowerment in health related decision-making, as well as engagement with a wider range of health knowledge that extends from personal health management to the social determinants of health.” Nutbeam [43], therefore, argues that thinking of health literacy in this regard has crucial ramifications, not only for changing peoples’ lifestyles and comprehension of prescription medication but also for improving peoples’ knowledge and their capability to act.

Conceptually, Nutbeam’s typologies, which have actions in health promotion such as education and social organization as their precursor, have a number of anticipated positive consequences across levels. In this study, we draw on Chinn and McCarthy’s [37] and Nutbeam’s model [41] to explore functional, communicative, and critical health literacy regarding foodborne diseases among the urban poor in Accra, Ghana. As recommended by Nutbeam [43], for examining communicative and critical health literacy, instruments were developed that assess oral literacy and societal abilities fundamental to negotiation and advocacy. This study is important because it will provide baseline information about the individual in an urban poor settlement’s capability to act and the social skills to advocate for themselves regarding foodborne diseases.

## Methods

### 2.1. Study area

James Town is a community in the Ashiedu Keteke sub-metropolitan area in the city of Accra, Ghana. The neighborhoods that comprise the community are Sempe, Akanmaadzen, and Ngleshie. Although it is a formal settlement, it fits the United Nations definition of slum, especially regarding persons per room, housing materials, sanitation facilities availability, and garbage disposal [44]. It is estimated that the room occupancy rate of the community is 7–9 persons per room. This community was chosen for this study for two reasons: (1) there are reports of high prevalence of foodborne diseases such as cholera, diarrhea, as well as typhoid fever, and the sub-metropolitan area has been labeled a cholera endemic community [45]; (2) the environmental conditions of the community make it susceptible to foodborne diseases incidence. Most of the community’s households do not have private sources of water, and there are just a few public toilet facilities [46] which lead to the practice of open defecation. An environmental assessment of the area has described it as unhygienic and unclean. Environmental sanitation is mainly poor, partly due to improper waste dumping [47].

### 2.2. Sample size, sampling, and research instruments

In 2010, the population of James Town was 15,508. The population was projected using the exponential population projection method (growth rate 2.2%; *t* − 7 years), and an estimated total population of 18,089 was determined. Epi Info 7 software was used to determine our sample size of 377 given the following power calculation statistics: confidence level = 95%; confidence interval (limit) = 5%; expected frequency (effect size) = 50%; design effect = 1; and cluster = 1. Ten percent more was added and 414 households were sampled. Out of these, a total of 401 individuals were administered the survey.

A mixed-method approach was used for this study. A survey questionnaire and in-depth interviews were administered. Chin and McCarthy ([37] p.252) recommended that quantitative instruments should be complemented by qualitative methods “to derive a more nuanced and contextualized understanding of health literacy as a social practice, rather than a set of predefined skills responsive to quantitative measurement and analysis.” The survey instrument was divided into sections: (1) closed-ended questions that elicited information on basic socio-demographic variables such as sex, age, education level, educational attainment, marital status, and employment status; (2) self-reported items (nine) on health literacy regarding foodborne diseases with each item having the response options “not at all,” “rarely,” “sometimes,” and “often.” Three questions each were developed that explored functional literacy, interactive/communicative and critical health literacy. The self-rated health statuses of respondents were elicited by using self-reporting of subjective health assessment. Specifically, respondents rated their health on a scale of 1 (poor health) to 5 (excellent health), which were later recoded into “poor,” “fair,” and “good.” Please see Appendix for questionnaire information.

Out of the total sample of 401 respondents, 30 individuals were selected for the in-depth interviews. The instrument guideline for the in-depth interviews was open-ended questions that probed for explanations and descriptions to the respondents’ (1) need for help with reading or comprehending information provided by medical personnel about foodborne diseases, (2) oral and interactive skills with medical professionals, and (3) attitudes and desire to seek information about foodborne diseases and be analytical about such information. For the research participants, foodborne disease was explained as “a disease caused by consuming contaminated food or drink including water.”

### 2.3. Analyses of data

The analyses of the quantitative data consisted of descriptive statistics, reliability analyses, validity analyses, analysis of variance (ANOVA), chi-square tests, and ordinal logit regression. We begin with a description of the socio-demographic characteristics of the respondents. Cronbach alpha coefficients were computed to assess the internal consistency of the scale of the entire instrument used and its subscales. Subsequently, a principal component analysis with varimax rotation was conducted to assess the factor structure of the scale. Health literacy scores were computed, and the relationship between health literacy, individual characteristics, and health status using ANOVA and ordinal logit regression models was then assessed.

#### 2.3.1. Reliability analyses

Reliability analyses were conducted to examine the internal consistency of the scale. A satisfactory Cronbach alpha value of 0.744 was achieved for the total scale of nine items. With regard to the subscales, the Cronbach alpha for the three items measuring functional health literacy was adequate (0.628), while the value for the three items measuring interactive health literacy was very good (0.830). The measure for the critical health literacy items achieved an adequate value of 0.625.

#### 2.3.2. Validity analyses

To examine content validity with regard to the fundamental structure of the major factors, a principal component analysis with varimax rotation was conducted. In view of the fact that the items were measuring the three typologies of health literacy, this analysis was to enable the assessment of whether each instrument item is loaded onto factors as desired. The analysis showed three factor loadings that were extracted with eigen values greater than 1. The first component had an eigen value of 3.16, and the second and third components had eigen values of 1.56 and 1.01, respectively; the three components accounted for 64% of the total variance. After the rotation, the interactive health literacy items all loaded onto factor 1, and the critical health literacy questions all loaded onto the second factor. The functional literacy questions loaded onto factor 3 (see Additional file 1).

#### 2.3.3. Univariate and multivariate analyses

ANOVA tests were used to examine bivariate relationships between the demographic variables (sex, age, educational level, educational attainment, marital status, employment status, religious affiliation, income) and health literacy scores. Ordinal logit regression models were run to assess whether health literacy were associated with health status. A main effect term (model 1) with health literacy and sex (the independent variables with the strongest of bivariate association with health status given an alpha value of 0.05) was built; then, an all two-way interaction terms with the other independent variables (models 2 and 3) was built, and, finally, a main effect term with health literacy and all the demographic characteristics that were significantly associated with self-rated health at the bivariate stage (model 4) was built. The model diagnostics suggests that the data fits the model well. That is, the assumption underpinning the proportional odds statistics have not been violated. Additionally, the multicollinearity analyses showed that none of the variance inflation factor values exceeded 10.

### 2.4. Qualitative data analyses

On the in-depth interview questions, a thematic analysis was carried out along the lines of the explanations given by respondents for their functional, interactive, and critical health literacy skills, thereby enabling deeper understanding. Transcribed data texts were read for emerging themes within the context of the study aims. Reading and re-reading of transcripts uncovered categories and associated concepts. Therefore, a method of triangulation was adopted, which is not simply meant to validate the survey responses, but to irradiate complementary facets of health literacy. The patterns that are important for the description of the quantitative results on self-reported reading, comprehension, writing, oral literacy, and societal abilities fundamental to negotiation and advocacy were appreciated. The similarities and differences across the health literacy typologies were depicted, as well as ensuring consistency. This is important primarily because qualitative data analysis is subjective and requires meticulousness and due diligence [48]. The patterns in the responses for each of the typologies of health literacy were identified and described as part of the results. Essentially, thematic analyses were used in this study as a *contextualist* technique, which appreciates how research participants make meaning of their health literacy experiences and how the broader cultural environment may influence those meanings.

## 3. Results

### 3.1. Characteristics of the study participants

As shown in Table 1, 60% of the respondents were female and nearly another 60% had junior high school level of education or lower, whereas about 42% were single, 38% reported that they were married, and 7% in consensual unions. While 7% indicated they had “poor” health status, 47% and 46% reported to have “fair” and “good” health, respectively.

**Table 1:** Characteristics of the study participants

Variables	Valid frequency (*N*)	Valid percent (%)
Sex		
Female	242	60.3
Male	159	39.7
Educational level		
No education	38	9.5
Primary	49	12.2
Junior high school	152	37.9
Senior high school	132	32.9
Tertiary	30	7.5
Educational attainment		
No education	37	9.2
Primary not completed	32	8.0
Primary completed	17	4.2
Junior high not completed	49	12.2
Junior high completed	90	22.4
Senior high not completed	73	18.2
Senior high completed	70	17.5
Tertiary not completed	8	2.0
Tertiary completed	25	6.2
Marital status		
Single	167	41.6
Consensual union	29	7.2
Divorced	32	8.0
Separated	22	5.5
Married	151	37.7
Employment status		
Unemployed	105	26.2
Employed	296	73.8
Religious affiliation		
No religion	96	23.9
Christian	274	68.3
Muslim	27	6.7
Traditionalist	4	1.0
Self-rated health		
Poor	27	6.7
Fair	189	47.1
Good	185	46.1

### 3.2. Health literacy scores, demographic characteristics, and health status

Regarding the scores on the health literacy skills items, while about 40% of the respondents reported not to require help when they are given information on foodborne diseases to read by a doctor, nurse, or pharmacist, almost 22 and 27% said they needed help “sometimes” and “often,” respectively. With respect to the interactive/communicative health literacy items, 20 and 56% of the respondents indicated that they “sometimes” and “often,” respectively, provide all the information needed for their own help when they talk to a doctor or nurse about foodborne diseases. Age, sex, employment status, and overall health literacy were found to be significantly associated with health status. Results from ANOVA examining the relationship between individual demographic characteristics (including sex, age, educational level, religious affiliation, and marital status) and health literacy only showed significant association between the following: functional health literacy and educational level, as well as critical health literacy score and marital status. From Table 2, under functional health literacy, we observed a statistically significant difference between the group with no education on the one hand and the groups with senior high school and tertiary education on the other hand. The differences in the means between primary education on the one hand and senior high school and tertiary on the other hand were statistically significant as well. With respect to critical health literacy and marital status, respondents who reported to be single are statistically different from those who reported their marital status as consensual union.

**Table 2:** Univariate analysis of variance of the relationship between demographic characteristics and health literacy scores: multiple comparisons (post hoc tests)

Functional health literacy				Critical health literacy			
(I) Educational level	(J) Educational level	Mean difference (I-J)	*P* value	(I) Marital status	(J) Marital status	Mean difference (I-J)	*P* value
No education	Primary	− 0.0601	1.000	Single	Consensual union	1.8102	0.032
	Junior high	0.7186	0.472		Divorced	0.1546	0.999
	Senior high	1.6763	0.001		Separated	0.3221	0.992
	Tertiary	1.7378	0.022		Married	0.1534	0.993
Primary	No education	0.0601	1.000	Consensual union	Single	− 1.8102	0.032
	Junior high	0.7788	0.271		Divorced	− 1.6555	0.244
	Senior high	1.7364	0.000		Separated	− 1.4881	0.463
	Tertiary	1.798	0.008		Married	−1.6568	0.068
Junior high	No education	− 0.7186	0.472	Divorced	Single	− 0.1546	0.999
	Primary	− 0.7788	0.271		Consensual union	1.6555	0.244
	Senior high	0.9577	0.006		Separated	0.1674	1.000
	Tertiary	1.0192	0.201		Married	− 0.0013	1.000
Senior high	No education	− 1.6763	0.001	Separated	Single	− 0.3221	0.992
	Primary	− 1.7364	0.000		Consensual union	1.4881	0.463
	Junior high	− 0.9577	0.006		Divorced	− 0.1674	1.000
	Tertiary	0.0615	1.000		Married	− 0.1687	0.999
Tertiary	No education	− 1.7378	0.022	Married	Single	− 0.1534	0.993
	Primary	− 1.798	0.008		Consensual union	1.6568	0.068
	Junior high	− 1.0192	0.201		Divorced	0.0013	1.000
	Senior high	− 0.0615	1.000		Separated	0.1687	0.999

Other independent variables controlled for include sex, age, and religious affiliation

In model 1 (Table 3), only the overall health literacy variable was significantly associated with self-rated health (0.041; *p* < 0.05). In model 2, we found significant interaction terms between health literacy and sex. Specifically, the interaction effects indicated ordered log-odds of 0.051 (*p* < 0.05) for overall health literacy and males. The interaction terms specified in model 3 for overall health literacy and individual characteristics that were significantly related with health status at the bivariate stage did not yield any statistically significant relationships. Model 4 shows that overall health literacy and employment status are significantly associated with self-rated health. The coefficient (0.034; *p* < 0.05) for overall health literacy is indicative of higher rating for self-rated health. For employment status, respondents who were unemployed had a coefficient of − 0.487, *p* < 0.05, which means the unemployed had a lower rating on the subjective health assessment.

**Table 3:** Ordinal logistic regression of self-rated health status

Variables	Categories	Model 1	Model 2	Model 3	Model 4
		Ordered log-odds	Ordered log-odds	Ordered log-odds	Ordered log-odds
Self-rated health	Poor	− 2.124***	− 1.973***	− 2.274***	− 2.212***
	Fair	0.668*	0.818**	0.670*	0.731
Overall health literacy	Health literacy	0.041*			0.034*
Sex	Female	− 0.279			−0.306
	Male (ref)				
Sex and overall health literacy	Female×health literacy		0.034	− 0.019	
	Male×health literacy		0.051*	0.000	
Health literacy and age				0.001	
Health literacy and employment status	Health literacy and unemployed			0.001	
Health literacy and employment status	Health literacy and employed			0.027	
Age					0.009
Employment status	Unemployed				− 0.487*
	Employed (ref)				

***P < 0.001; **P < 0.005; *P < 0.05

### 3.3. In-depth interviews

The results of the in-depth interviews are presented as themes and sub-themes for each of the typologies of health literacy. The explanations for responses on functional literacy—the need for help with reading or comprehending information provided by medical personnel about foodborne diseases—yielded three main themes: factors associated with perception regarding medical personnel, individual personal abilities, and presence or absence of social capital. Regarding factors associated with perception on medical personnel, respondents who mostly required help with reading, comprehension, and completion of hospital documents indicated that the information they are provided is purely medical and, therefore, with no or limited educational status, they needed doctors and nurses to help them. Factors related to individual abilities were with regard to the respondents’ capability to write, read, and level of comprehension. A participant explained his positive functional ability as follows:

I can do it myself. I can read and write and understand everything because I went to Junior High School. I developed how to write my name and other things so I do not need help to fill forms or to understand any information [Male, aged 37]

Social capital was another important theme regarding functional health literacy. Some respondents who needed some form of help with comprehending health information or completing hospital forms relied on neighbors, friends, and family. But it is worth noting that not all got help in this regard, even if they needed it.

Explanations on responses for interactive/communicative literacy—their oral and interactive skills with medical professionals—generated two themes: (1) factors associated with perceptions regarding medical personnel and (2) quest to remain healthy. Concerns emerging from factors associated with perceptions on medical personnel include the following: perceived impatience of doctors and nurses, perceived professional gap between medical staff and clients/patients, and perceived unfriendliness of medical staff. Respondents suggested that the exercise of their interactive skills is predicated on whether or not they think of the medical professional as having enough patience to discuss concerns and explain ambiguous information. A respondent described and speculated the perceived unfriendly demeanor of some doctors:

“Sometimes I ask [questions], and sometimes I don’t ask [questions]. I first look at the person [that is, the doctor or nurse] before I talk. Whether he or she is receptive or not... Some doctors and nurses are not friendly at all and might insult you…” [Female, aged 59]

Other respondents who saw the need to communicate more effectively with doctors and nurses on foodborne diseases mainly indicated their quest to remain healthy. They reported that the opportunity to be with a doctor or nurse should not be wasted, and there is the need to ask all questions one has as well as provide all the needed information. By so doing, one is likely to (1) avoid making mistakes that caused the sickness from a foodborne disease, (2) get the right prescription, and (3) understand all the causes of various foodborne diseases.

The themes emerging for critical health literacy—their attitudes and desire to seek information about foodborne diseases and be analytical about such information—are the following: perceived sacrosanct nature of expert opinion, factors related to personal life, and reliance on mass media. In relation to whether the respondents thought carefully about whether information on foodborne diseases make sense in a particular way, some respondents did not see the need to think about it at all because the information coming from the “experts” was perceived as sacrosanct. Others thought that it was necessary to think about health information and question doctors’ decision. While some pointed to mistakes made by medical staff that has resulted in the demise of relatives and friends, others simply said that medical personnel cannot be fully trusted. On factors related to personal life, respondents who sometimes and often carefully think about information on foodborne diseases and question doctors’ advice intimated that personal life experience, such as having had the disease previously or a family member or friend’s experience, is enough to compel critical examination of health information. On the other hand, participants who do not carefully think about foodborne disease information provided to them mentioned personal life issues like work schedule and busy schedule with house chores as reasons for not paying attention to health information. The sub-theme of reliance on mass media was mainly in response to whether or not respondents deliberately seek information about foodborne diseases. The radio and television were the sources of information on foodborne diseases.

## 4. Discussion

This study found associations between higher health literacy and demographic variables such as higher education level and marital status (respondents who reported to be single and those in a consensual union). Specifically, although functional literacy is relatively low in James Town, we found a positive association between educational level and functional health literacy. That is, higher education is associated with higher functional health literacy. Additionally, the study found a direct positive relationship between health literacy and health status, and demographic variables such as age, sex, employment status, and overall health literacy were significantly associated with self-rated health status. The association between health literacy and self-rated health varies significantly between the sexes. Therefore, males with higher health literacy had higher rating on self-rated health in comparison to their female counterparts.

A tool was developed to understand three typologies of health literacy, namely functional, communicative/interactive, and critical health literacy among the urban poor. The reliability and validity analyses of the instrument showed satisfactory indices. The internal consistency measures of the scale and subscales were good, although a couple of the subscales were adequate. Subsequent measures may have to initially engage the community and its opinion leaders for improving the nature of questions as exemplified by Chinn and McCarthy [37] in their project design for All Aspect of Health Literacy Scale. This may help achieve excellent internal consistency across all subscales.

Regarding our findings on education and health literacy, the means score of functional health literacy increased with educational level. That is, the higher one’s educational level, the higher one’s functional health literacy score, in consonance with Bann et al.’s [26] findings. Therefore, those with senior high school and tertiary level education were more likely not to require help in completing official documents in the hospital and were more likely not to need someone to help them comprehend information given to them by the doctor, nurse, or pharmacist on foodborne diseases. This finding is supported and further illuminated by the in-depth interview results, especially with the theme “individual abilities”—one’s ability to read, write, and comprehend text, which is a function of educational level. This is consistent with the findings in the literature that people with lower educational level show lower health literacy skills compared with people with higher education [27] partly due to limited comprehension of health information as well as inadequate understanding of medical instructions [29]. This is further testament to the affirmation that low health literacy is both a personal inadequacy issue as well as indicative of underprivileged social status [27].

The in-depth interview results on personal abilities and respondents’ perception about medical professionals are critical in understanding health literacy. Clearly, respondents who (1) trust their doctors/nurses and (2) perceive their doctors and nurses as friendly, willing to help, patient, and not condescending were more willing to engage their medical professional. While we do not intend to necessarily make policy prescriptions on this exploratory study, this finding (1) shows the need for health professionals to be proficient at all times and (2) elucidates Chinn and McCarthy’s [37] suggestion for health care providers to be cautious so as not to assume uniformity in health literacy among community members. Although we did find communicative and critical health literacy scores to be significantly different across educational levels, on the individual measures for critical health literacy, respondents with higher education indicated the possibility of questioning their doctor or nurse’s advice on foodborne diseases based on their own research/findings, as illustrated by both quantitative and qualitative results. Therefore, the pathways by which health literacy in the area of foodborne diseases may mediate educational level and foodborne diseases outcome are still a fertile research ground worthy of further exploration.

The self-rated health of respondents was significantly associated with overall health literacy after adjusting for demographic characteristics. Specifically, self-rated health status was positively related to health literacy as illustrated in other studies [28, 29]. That implies that the more individuals are able to give doctors and nurses (1) all the information they need to help them, (2) ask all the questions they need to ask, and (3) make sure anything they do not understand is explained to them, the more likely it is for better health outcomes. Additionally, the more people like to find out lots of different information about their health regarding foodborne diseases, think carefully about whether health information on foodborne diseases makes sense, and question their doctor or nurse’s advice on foodborne diseases based on their own research/findings, better health ratings are expected. Such skills are needed to function effectively in health care settings [24] and are more likely to stretch from personal health management to social determinants of health [42], thereby positively influencing health status. The overall health literacy variable assessed oral literacy and societal skills fundamental to negotiation and advocacy [43], and the results seem to suggest that individuals with more of such skills have better health status because they employ such abilities during their interactions with medical personnel.

### 4.1. Limitations

This study has a few limitations worth noting. First, the functional, interactive, and critical health literacy measures designed for the individual level analyses are inadequate to expound the complexities in the linkage between health literacy and health outcomes cognizant of the macro level factors inherent to health literacy, which include the healthcare system and other social and environmental elements [31]. Secondly, health literacy may be significantly associated with unobserved variables that were not controlled for in our models. These may include ethnicity, type of employment, and specific food variables (access to food—availability of household food, affordability of food vended, and the extent of patronage of different food sources). Thirdly, while the use of subjective health status is a universally accepted measure of health, an objective assessment of the health status of respondents with specific emphasis on foodborne disease may have shown a more direct link between health literacy on foodborne diseases and the prevalence of foodborne illnesses in James Town.

## 5. Conclusion

Ghana experiences high incidence of foodborne diseases. An urban poor settlement known to have high prevalence of foodborne diseases was chosen for this study. In this paper, we have illustrated that, in general, health literacy is demographically differentiated and related with health status in James Town. An improvement in functional, communicative, and critical health literacy in the area of foodborne diseases has the potential of improving the well-being of the people. This study has evidently shown the positive linkages between higher educational level and functional health literacy and has also demonstrated the direct positive relationship between health literacy and health status controlling for covariates. Given the perceptions of individuals regarding interactions with health practitioners, health professionals during dealings with clients should not assume uniformity of knowledge or actions among the poor.

### Additional file

Additional file 1: Instrument Item Scores and Principal Component Analysis Factor Loadings. (DOCX 14 kb)

## Appendix

The questions on the health literacy typologies are as follows:

Functional literacy:

- How often do you need someone to help you when you are given information on foodborne diseases to read by your doctor, nurse, or pharmacist?
- When you need help to understand information about foodborne diseases, can you easily get someone to assist you?
- Do you need help to fill in official documents at the hospital or clinic?

Interactive/communicative literacy:

- When you talk to a doctor or nurse about foodborne diseases, do you give them all the information they need to help you?
- When you talk to a doctor or nurse about foodborne diseases, do you ask all the questions you need to ask?
- When you talk to a doctor or nurse about foodborne diseases, do you make sure they explain anything that you do not understand?

Critical literacy

- Are you someone who likes to find out lots of different information about your health regarding foodborne diseases?
- How often do you think carefully about whether health information on foodborne diseases makes sense in a particular situation?

Are you the sort of person who might question your doctor or nurse’s advice on foodborne diseases based on your own research/findings?

## Abbreviations

ANOVA: Analysis of variance
DALYs: Disability adjusted life years
REALM-R: Rapid estimate of adult literacy in medicine
TOFHLA: Test of functional health literacy in adults
WHO: World Health Organization
