# Supplementary material for: Self-reported functional, communicative, and critical health literacy on foodborne diseases in Accra, Ghana
Source: Trop Med Health. 2018 May 15;46:15. doi: 10.1186/s41182-018-0097-6 (PMC5952472; doi:10.1186/s41182-018-0097-6)
Supplement: Supplementary file 1 — Instrument Item Scores and Principal Component Analysis Factor Loadings. (DOCX 14 kb) [file 41182_2018_97_MOESM1_ESM.docx]

Table S1: Instrument Item Scores and Principal Component Analysis Factor Loadings

| Instrument Items | Item scores | Principal Component Analysis | | |
| --- | --- | --- | --- | --- |
|  |  | Factor loadings | | |
|  | (Means and SD) | Factor 1 | Factor 2 | Factor 3 |
| How often do you need someone to help you when you are given information on foodborne diseases to read by your doctor, nurse, or pharmacist? | 2.3524 (1.25) | 0.111 | -0.025 | 0.807 |
| When you need help to understand information about foodborne diseases, can you easily get someone to assist you? | 2.6132 (1.23) | 0.143 | 0.134 | 0.675 |
| Do you need help to fill in official documents at the hospital or clinic? | 2.5817 (1.30) | 0.047 | 0.006 | 0.759 |
| When you talk to a doctor or nurse about foodborne diseases, do you give them all the information they need to help you? | 3.1948 (1.10) | 0.829 | 0.107 | 0.187 |
| When you talk to a doctor or nurse about foodborne diseases, do you ask all the questions you need to ask? | 3.0057 (1.12) | 0.833 | 0.306 | 0.12 |
| When you talk to a doctor or nurse about foodborne diseases, do you make sure they explain anything that you do not understand. | 3.2493 (1.06) | 0.823 | 0.178 | 0.068 |
| Are you someone who likes to find out lots of different information about your health regarding foodborne diseases? | 2.5473 (1.24) | 0.188 | 0.759 | -0.007 |
| How often do you think carefully about whether health information on foodborne diseases makes sense in a particular situation? | 2.937 (1.10) | 0.318 | 0.696 | 0.011 |
| Are you the sort of person who might question your doctor or nurse’s advice on foodborne diseases based on your own research/findings? | 2.1977 (1.23) | 0.053 | 0.736 | 0.105 |

Rotation Method: Varimax with Kaiser Normalization
